# Supplementary material for: The Deadly Toxin Arsenal of the Tree-Dwelling Australian Funnel-Web Spiders
Source: Int J Mol Sci. 2022 Oct 28;23(21):13077. doi: 10.3390/ijms232113077 (PMC9658043; doi:10.3390/ijms232113077)
Supplement: Supplementary file 1 [file ijms-23-13077-s001.zip › ijms-1954014-supplementary.pdf]

Supplementary Material

Supplementary Figure S1. TICs of defensive venom collected from female individual specimens (two for each species, on left and right panels) of FWS. The venom profiles appear consistent within each species, exhibiting only minimal intraspecific variation (mostly related to variation in intensity of some components). From the two species of tree-dwelling FWS, the venom profile of *H. cerberea* resembles more ground dwelling species like *H. infensa*, being highly complex and with components eluting around the same time, whereas the venom profile of *H. formidabilis* appears unique, being more streamlined.

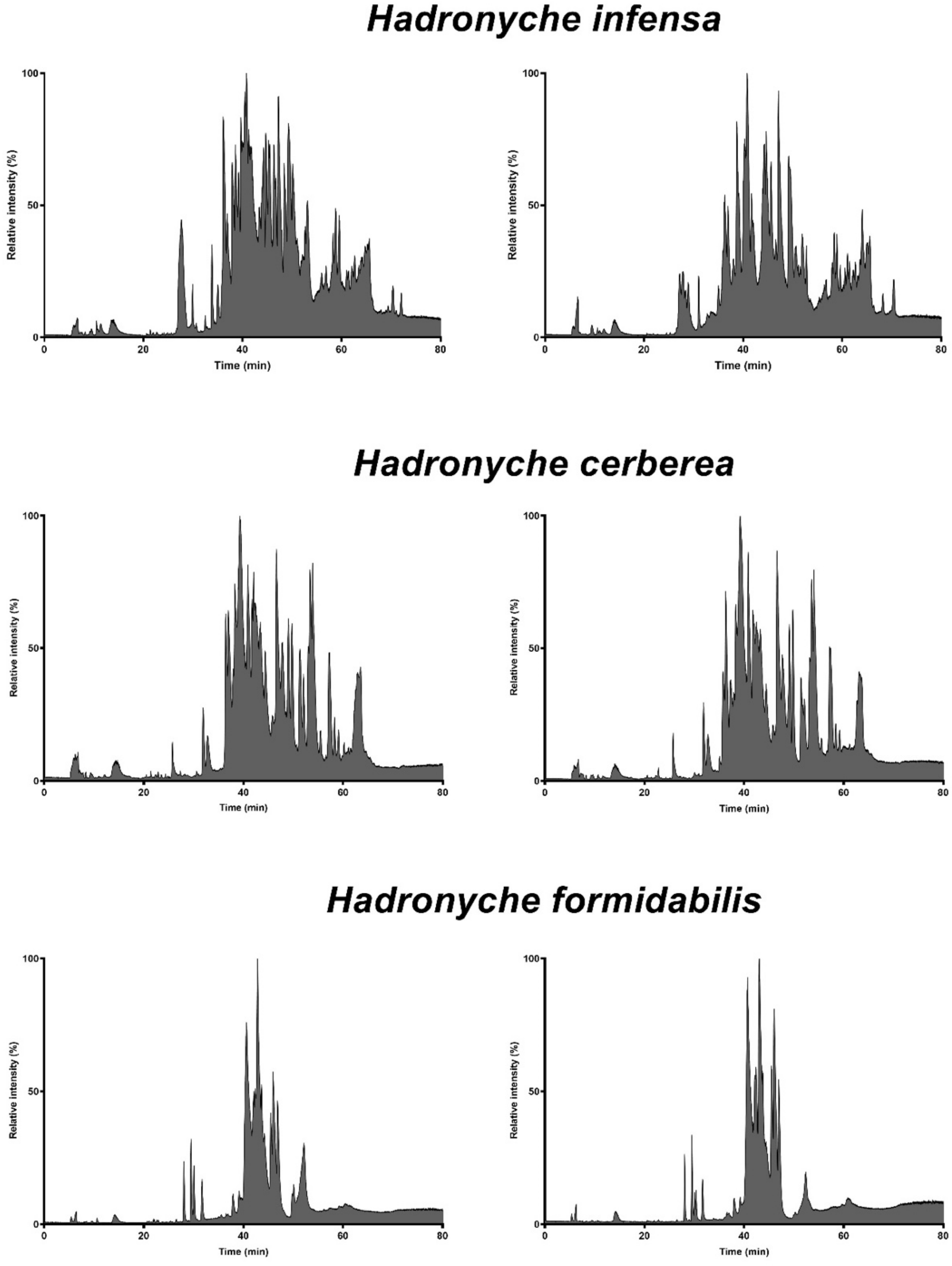

<u>Supplementary Figure S2.</u> The pharmacological activities of the FWS crude venoms on mammalian voltage-gated sodium (Na_V) and calcium (Ca_V) channel subtypes. Tests were performed at (in ng/ml) (A) 250, (B) 25 and (C) 2.5 concentration of venom and using fluorescence-imaging cellular assays in endogenously expressed human (h) Cav channels or recombinantly expressed rat (r) or human (h) Nav channels. The enhanced responses above 100% activation of the control without venom were considered, and cut-off represented by the red dashed lines. Data are represented by mean ± SD from n = 2 specimens of *H. cerberea*, *H. infensa* or *H. formidabilis*, and by n = 1 specimen of *A. robustus* (female) for each tested target. The area under the curve (AUC) was considered for these analyses.

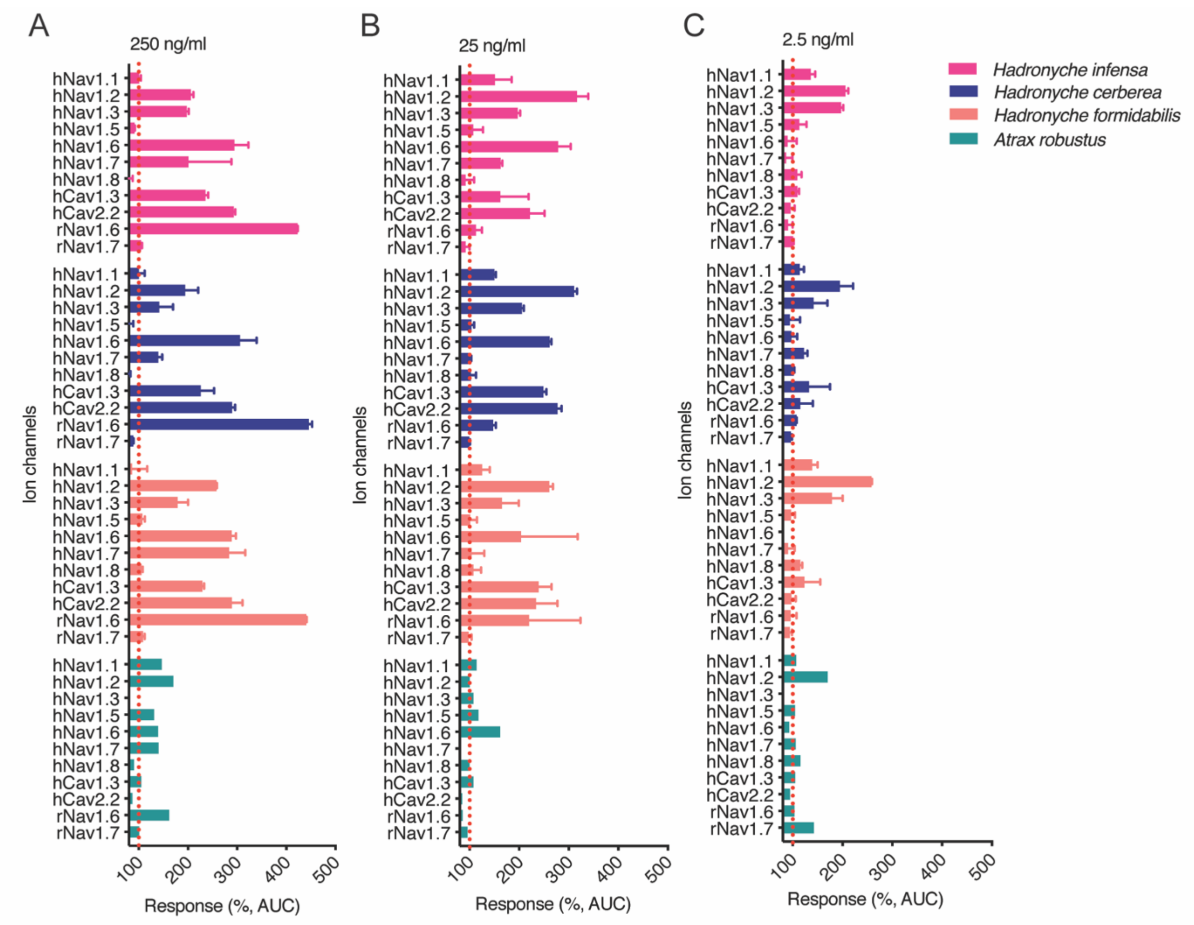

<u>Supplementary Figure S3</u>. Maximum responses of ion channels elicited by assay controls. The standard deviation from the activation controls for each ion channel subtype assayed were described as reference for the results in Figure 6. Data are from n = 3 (Cav assays) or n = 6-9 (Nav assays), and represented as the mean ± SD.

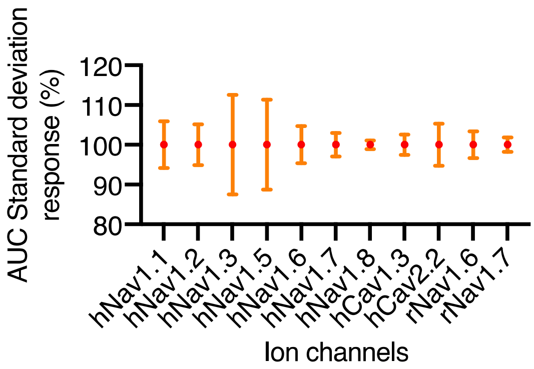

Supplementary Table S1: Catalogue of Superfamilies (SF) identified from the transcriptome of the venom gland of the tree-dwelling species *Hadronyche cerberea* and *Hadronyche formidabilis*. This supplementary material is provided as excel formatting.

Supplementary Table S2: Peptides and proteins comprised in the milked venom of tree-dwelling FWS revealed by shotgun proteomic analysis. We identified 99 peptide/protein sequences in the venom of *H. formidabilis* (A) and 127 peptide/protein sequences in the venom of *H. cerberea* (B). This supplementary material is provided as excel formatting.

Supplementary Table S3: Molecular Evolution of Tree-dweller vs Ground-dwelling Spider Toxin Superfamilies (table with sites and properties under selection). A detailed legend for the employed abbreviations is provided below.

**PAML (M8)**			**TreeSAAP**				**MEME sites**
**Positive selected site**	**AA**	**Post mean ± SE for ω**	**Conservative changes in A.A properties**		**Radical changes in A.A properties**		
			**Chemical**	**Physical**	**Chemical**	**Physical**	
***Hadronyche formidablis*: True Tree Dweller**							
**SF13**		**ω: 0.64**					**1**
-	-	-					
**SF9**		**ω: 0.62**					**19**
-	-	-					
**SF10**		**ω: 2.59**		**2**		**3**	**0**
84	V	9.041 ± 1.766		pH_i		α_m	
85	C	9.033 ± 1.786		pH_i		α_m	
100	A	9.026 ± 1.806				R_a	
-	-	-					
**SF4**		**ω: 0.98**		**16**		**51**	**45**
94	S	3.729 ± 0.445		N_s, M_v, H_{nc}, V^o		P_c, h, α_n, P	
95	D	3.726 ± 0.453		N_s, M_v, V^o		P_c, h, α_n, P	
101	T	3.728 ± 0.446				P_c, C_a, h, α_c	
102	A	3.728 ± 0.447				P_c, C_a, h, α_c, E_{sm}	
105	G	3.729 ± 0.446				P_c, C_a, H_{nc}, α_c, E_{sm}	
106	I	3.727 ± 0.451				P_c, C_a, h, H_{nc}, α_c, E_{sm}	

108	V	3.714 ± 0.488				P_c, C_a, h, E_l, H_{nc}, E_{sm}	
114	S	3.658 ± 0.616		P_α, M_v, V^o		P_c, h, H_{nc}, α_n, E_{sm}, P	
115	A	3.723 ± 0.462		P_α, M_v, V^o		P_c, h, H_{nc}, α_n, E_{sm}, P	
118	C	3.723 ± 0.461		P_α, V^o, E_{sm}		P_c, h, H_{nc}, α_n, P	
**SF26**		**ω: 1.55**		**14**		**53**	**6**
62	L	5.119 ± 0.560		p		h, α_n	
64	G	5.081 ± 0.687		p		K^o, h, α_n	
65	S	5.055 ± 0.759		p		K^o, h, α_n	
67	C	4.934 ± 1.026		p		K^o, h, α_n	
68	A	5.120 ± 0.559		p		K^o, h, α_n	
69	R	5.107 ± 0.605				K^o, h, α_n	
70	T	5.088 ± 0.663				K^o, h, α_n	
72	S	5.098 ± 0.634				K^o, h	
73	A	4.976 ± 0.936		p		K^o, h	
75	S	5.115 ± 0.574		p		K^o, h, α_n	
76	G	5.062 ± 0.738				K^o, h, α_n	
80	N	5.093 ± 0.649		p		K^o, h, α_n	
82	C	5.119 ± 0.560		p		K^o, h, α_n	
83	Y	5.120 ± 0.559		p		K^o, h, α_n	
84	I	5.119 ± 0.561		p		K^o, h, α_n	
85	C	5.119 ± 0.559		p		K^o, h, α_n	
86	L	5.119 ± 0.559		p		K^o, h, α_n	
87	L	5.119 ± 0.559		p		K^o, h, α_n	
89	S	5.119 ± 0.560				H	
90	S	5.108 ± 0.600				H	
91	M	5.115 ± 0.574					

***Hadronyche cerberea*: Newly Adapted Tree Dweller**							
**SF13**		**ω: 0.20**					**0**
-	-	-					
**SF9**		**ω: 1.90**					**1**
78	G	9.419 ± 1.285					
79	N	9.111 ± 2.044					
**SF10**		**ω: 0.75**					**1**
-	-	-					
**SF4**		**ω: 2.85**		**19**		**72**	**18**
95	D	10.108 ± 1.841		C_a		B_l, P_c, h, α_m	
96	N	10.422 ± 0.712		C_a		B_l, P_c, h, α_m	
97	D	10.469 ± 0.238		C_a		B_l, P_c, h, α_m	
98	V	10.472 ± 0.167		C_a		B_l, P_c, h, α_m	
99	L	10.424 ± 0.700				B_l, h, α_m	
101	T	10.471 ± 0.193				B_l, P_c, α_m	
102	V	10.471 ± 0.202				B_l, P_c, α_m	
103	G	10.412 ± 0.780				B_l, P_c, h, α_m	
105	G	10.472 ± 0.166				B_l, P_c, α_m	
106	G	10.472 ± 0.165				B_l, P_c, h, α_m	
107	N	10.472 ± 0.165				B_l, P_c, h, α_m	
108	V	10.472 ± 0.165				B_l, P_c, h, α_m	
109	I	10.472 ± 0.167		*Ca*		B_l, P_c, h, α_m	
110	V	10.472 ± 0.165		*Ca*		B_l, P_c, h, α_m	

112	G	10.472 ± 0.165		*Bl, Ca*		B_l, P_c, h, α_m	
113	F	10.472 ± 0.177		*Bl, Ca*		B_l, P_c, α_m	
114	S	10.472 ± 0.165		*Bl, Ca*		P_c, h, α_m	
115	L	10.472 ± 0.173		*Bl, Ca*		P_c, h, α_m	
116	F	10.472 ± 0.167		*Bl, Ca*		P_c, h, α_m	
117	Q	10.472 ± 0.165		*Bl, Ca*		P_c, α_m	
118	C	10.472 ± 0.165		*Ca*		P_c	
119	W	10.472 ± 0.165					
120	G	10.151 ± 1.741					
**SF26**		**ω: 1.85**	**12**	**6**		**81**	**18**
61	K	6.238 ± 0.834	*pK'*			pH_i, α_n, R_a	
62	E	6.289 ± 0.675	*pK'*			K^o, pH_i, α_n	
63	N	6.284 ± 0.693	*pK'*			K^o, pH_i, α_n	
66	G	6.276 ± 0.724				K^o, α_n	
67	C	6.068 ± 1.232				K^o, α_n	
69	R	6.297 ± 0.647				K^o, h, α_n, E_{sm}	
70	T	6.296 ± 0.648				K^o, h, α_n, E_{sm}	
72	S	6.296 ± 0.648		α_m		K^o, h, α_n, E_{sm}	
73	A	6.187 ± 0.981		α_m		K^o, h, α_n, E_{sm}	
77	G	6.251 ± 0.807		α_m, P		K^o, h, α_n, E_{sm}, R_a	
79	V	6.295 ± 0.654		P		K^o, h, α_n, E_{sm}, R_a	
80	N	6.259 ± 0.782		P		K^o, h, α_n, E_{sm}, R_a	
81	E	6.270 ± 0.748	*pK'*			K^o, h, α_n, E_{sm}, R_a	
82	R	6.297 ± 0.647	*pK'*			K^o, h, α_n, E_{sm}, R_a	
83	Y	6.297 ± 0.648	*pK'*			K^o, h, pH_i, α_n, E_{sm}, R_a	
84	I	6.296 ± 0.651	*pK'*			K^o, h, pH_i, α_n	
85	C	6.297 ± 0.647	*pK'*			K^o, h, pH_i, α_n	

86	N	6.249 ± 0.819	pK'			K^o, h, pH_i, α_n	
87	L	6.296 ± 0.649	pK'			K^o, h, α_n	
88	E	6.296 ± 0.648	pK'			h, α_n	
89	S	6.297 ± 0.648	pK'			α_n	
90	S	6.296 ± 0.648				α_n	
91	M	6.296 ± 0.648				α_n	
92	C	6.295 ± 0.655				α_n	
**Ground Dwelling Spiders from Atracidae Family**							
**SF13**		**ω: 1.58**		**32**		**7**	**8**
95	G	7.548 ± 0.854		C_a			
96	A	7.540 ± 0.881		C_a			
97	w	7.581 ± 0.721		C_a		M_v	
98	Y	7.581 ± 0.720		C_a		M_v	
100	Q	7.582 ± 0.720		C_a, M_v, V^o, μ			
101	Q	7.567 ± 0.781		C_a, M_v, V^o, μ			
102	S	7.581 ± 0.720		C_a, M_v, V^o, μ			
103	S	7.582 ± 0.720		C_a, M_v, V^o, μ			
105	Q	7.582 ± 0.720		C_a, M_v, V^o, μ			
106	S	7.582 ± 0.720		C_a, M_v, V^o, μ			
107	T	7.582 ± 0.720		C_a, V^o, μ		M_v	
109	M	7.582 ± 0.720		μ		M_v	
110	G	7.581 ± 0.722				M_v	
111	M	7.582 ± 0.720				M_v	
112	F	7.581 ± 0.720				M_v	
114	K	7.582 ± 0.720					
115	C	7.465 ± 1.111					

**SF9**		**ω: 0.64**					**15**
-	-	-					
**SF10**		**ω: 0.94**		**14**	**9**		**8**
58	V	3.304 ± 0.624		P_a, F, α_n, R_a			
80	S	3.324 ± 0.592			pK'		
81	C	3.380 ± 0.469			pK'		
82	T	3.306 ± 0.617			pK'		
87	A	3.370 ± 0.491			pK'		
93	L	3.340 ± 0.555		P_a, R_a			
97	L	3.304 ± 0.622		P_a, α_m	pK'		
98	L	3.386 ± 0.453		P_a, α_m	pK'		
99	G	3.272 ± 0.680		P_a, α_m	pK'		
101	A	3.386 ± 0.452		α_m	pK'		
102	L	3.384 ± 0.457		α_m	pK'		
**SF4**		**ω: 1.42**		**59**		**327**	**73**
49	Q	4.750 ± 0.013		P_a, V^o, α_m		α_c, R_a	
85	E	4.738 ± 0.217		N_s, c, pH_i, H_p		B_l, B_r, P_c, K^o, h, F, M_v, M_w, V^o, α_c, α_n, μ, E_{sm}, R_a, H_t	
86	T	4.750 ± 0.000		N_s, c, pH_i		B_l, B_r, P_c, K^o, h, F, M_v, M_w, V^o, α_c, α_n, μ, R_a, H_p, H_t	
88	F	4.749 ± 0.064		N_s, c, pH_i		B_r, P_c, K^o, h, F, M_w, H_{nc}, α_c, α_n, R_a, H_p, H_t	
90	S	4.750 ± 0.035		N_s, c		B_r, P_c, K^o, h, M_w, H_{nc}, α_c, α_n, R_a, H_p, H_t	
91	D	4.725 ± 0.307		N_s		B_r, P_c, K^o, h, M_w, H_{nc}, α_c, α_n, R_a, H_p, H_t	

93	T	4.750 ± 0.007		c		B_r, P_c, K^o, h, F, M_v, M_w, H_{nc}, V^o, α_c, α_n, μ, H_p, H_t	
94	S	4.750 ± 0.000		c		B_r, P_c, K^o, h, F, M_v, M_w, H_{nc}, V^o, α_c, α_n, μ, H_p, H_t	
95	N	4.741 ± 0.185		c, P_r		B_r, K^o, h, F, M_v, M_w, H_{nc}, V^o, α_c, α_n, μ, E_{sm}, H_p	
98	A	4.750 ± 0.000		P_r, p		B_l, B_r, K^o, C_a, h, F, M_v, M_w, V^o, μ, E_{sm}, H_p, H_t	
99	L	4.750 ± 0.012		P_r, p		B_l, B_r, K^o, C_a, h, F, M_v, M_w, V^o, μ, E_{sm}, H_t	
100	N	4.750 ± 0.023		P_a, P_r, p		B_l, B_r, K^o, C_a, h, F, M_v, M_w, V^o, μ, E_{sm}, H_t	
101	T	4.750 ± 0.000		P_a, P_r, p		B_l, C_a, h, F, M_v, M_w, V^o, μ	
102	V	4.750 ± 0.000		P_a, P_r, p		B_l, C_a, h, M_v, M_w	
103	G	4.750 ± 0.000		P_a, pH_i, P_r		Bl, C_a, M_v	
104	Q	4.750 ± 0.000		P_a, pH_i, P_r		h, M_v, H_{nc}, E_{sm}	
105	G	4.750 ± 0.001		P_a, pH_i, V^o, P_r		B_l, h, M_v, H_{nc}, E_{sm}, R_a	
106	L	4.750 ± 0.000		pH_i, V^o		B_l, h, M_v, H_{nc}, E_{sm}, R_a, H_p	
107	N	4.718 ± 0.349		pH_i		B_l, P_c, h, F, M_v, M_w, H_{nc}, V^o, μ, E_{sm}, R_a, H_p, H_t	
108	V	4.750 ± 0.000		pH_i		B_l, P_c, h, F, M_v, M_w, H_{nc}, V^o, μ, E_{sm}, R_a, H_p, H_t	
109	V	4.750 ± 0.000		pH_i		B_l, P_c, h, F, M_v, M_w, H_{nc}, V^o, μ, E_{sm}, R_a, H_p, H_t	
110	S	4.750 ± 0.000		pH_i		B_l, P_c, h, F, M_v, M_w, H_{nc}, V^o, μ, E_{sm}, R_a, H_p, H_t	
112	G	4.750 ± 0.008		pH_i		B_l, P_c, h, F, M_v, M_w, H_{nc}, V^o, μ, E_{sm}, R_a, H_p, H_t	

113	L	4.655 ± 0.605		pH_i		$B_l, P_c, K^o, h, F, M_v, M_w, H_{nc}, V^o, \alpha_n, \mu, E_{sm}, R_a, H_p, H_t$	
114	S	4.750 ± 0.023		pH_i		$B_l, P_c, K^o, h, F, M_v, M_w, H_{nc}, V^o, \alpha_n, \mu, E_{sm}, R_a, H_p, H_t$	
115	A	4.750 ± 0.006		pH_i		$B_l, P_c, K^o, h, F, M_v, M_w, H_{nc}, V^o, \alpha_c, \alpha_n, \mu, E_{sm}, R_a, H_p, H_t$	
116	F	4.729 ± 0.287		pH_i		$B_l, P_c, K^o, h, F, M_v, M_w, H_{nc}, V^o, \alpha_c, \alpha_n, \mu, E_{sm}, R_a, H_p, H_t$	
117	Q	4.750 ± 0.003		pH_i, H_t		$P_c, K^o, h, M_w, H_{nc}, \alpha_c, \alpha_n, R_a, H_p$	
118	C	4.750 ± 0.000		pH_i, H_t		$P_c, K^o, h, M_w, H_{nc}, \alpha_c, \alpha_n, R_a, H_p$	
119	W	4.750 ± 0.000		H_t		P_c, K^o, α_c, R_a	
**SF26**		**ω: 1.18**		**10**		**119**	**14**
51	R	2.783 ± 0.458				$B_l, K^o, h, \alpha_c, \alpha_n, R_a$	
61	K	2.725 ± 0.550		P_r		$B_l, h, E_l, F, \alpha_n, R_a$	
62	T	2.763 ± 0.494		p, μ		$B_l, K^o, h, E_l, F, \alpha_n, R_a$	
63	Y	2.766 ± 0.488		p, μ		$B_l, K^o, h, E_l, F, \alpha_n, R_a$	
67	C	2.710 ± 0.586		p		$Bl, K^o, F, \alpha_n, R_a$	
70	T	2.785 ± 0.454		p		K^o, h, α_n	
71	W	2.758 ± 0.503		p		$K^o, h, E_l, \alpha_n, R_a$	
72	S	2.782 ± 0.460		p		$B_l, K^o, h, E_l, \alpha_n, R_a$	
74	M	2.761 ± 0.496		p		$B_l, K^o, h, E_l, \alpha_n, R_a$	
77	G	2.721 ± 0.562				$B_l, K^o, h, E_l, \alpha_n, R_a$	
80	R	2.774 ± 0.475				h, E_l, α_c	
81	E	2.716 ± 0.561				h, E_l, α_c	
82	C	2.786 ± 0.452				$h, E_l, \alpha_c, \alpha_n$	
83	Y	2.784 ± 0.456				$h, E_l, \alpha_c, \alpha_n$	
84	I	2.723 ± 0.555				$h, E_l, \alpha_c, \alpha_n, R_a$	

85	C	2.744 ± 0.530				h, E_l, α_c, α_n, R_a	
86	A	2.750 ± 0.518				K^o, h, E_l, α_c, α_n, R_a	
87	V	2.786 ± 0.453				K^o, h, E_l, α_c, α_n, R_a	
88	E	2.771 ± 0.478				K^o, h, E_l, α_c, α_n, R_a	
89	S	2.731 ± 0.545				K^o, h, E_l, α_c, R_a	
90	S	2.755 ± 0.509				K^o, h, E_l, α_c, R_a	
91	M	2.786 ± 0.453				K^o, h, E_l, α_c, R_a	
92	C	2.778 ± 0.468				K^o, h, E_l, α_c, R_a	

Legend: α−helical tendencies (P_α); β-structure tendencies (P_β); Average # surrounding residues (N_s); Bulkiness (B_l); Buriedness (B_r); Chromatographic index (R_F); Coil tendencies (P_c); Composition (c); Compressibility (K^o); Equilibrium Const. – ionization, COOH (pK'); Helical contact energy (C_a); Hydropathy (h); Isoelectric point (pH_i); Long-range n.b. energy (E_l); Mean r.m.s. fluctuation displacement. (F); Molecular volume (M_v); Molecular weight (M_w); Normal. consensus hydrophobicity (H_{nc}); Partial specific volume (V^o); Polar requirement (P_r); Polarity (p); Power to be – C-term. α-helix (α_c); Power to be – middle, α-helix (α_m); Power to be – N-term., α-helix (α_n); Refractive index (μ); Sh.- & med.-range n.b. energy (E_{sm}); Solvent accessible reduct. ratio (R_a); Surrounding hydrophobicity (H_p); Thermodynamics transfer hydrophobicity (H_t); Total n.b. energy (E_t); Turn tendencies (P)

Legend (tabular format)

**Properties**	**Symbol**	**Property type**
Equil. Const. – ioniza., COOH	pK'	Chemical
Average # surrounding residues	N_s	Physical
Bulkiness	B_l	Physical
Buriedness	B_r	Physical
Chromatographic index	R_F	Physical
Coil tendencies	P_c	Physical
Composition	c	Physical
Compressibility	K^o	Physical
Helical contact energy/area	C_a	Physical
Hydropathy	h	Physical
Isoelectric point	pH_i	Physical
Long-range n.b. energy	E_l	Physical
Mean r.m.s. fluctuat. displace.	F	Physical
Molecular volume	M_v	Physical
Molecular weight	M_w	Physical
Normal. consensus hydrophob.	H_{nc}	Physical
Partial specific volume	V^o	Physical
Polar requirement	P_r	Physical
Polarity	p	Physical
Power to be – C-term. α-helix	α_c	Physical
Power to be – middle, α-helix	α_m	Physical
Power to be – N-term., α-helix	α_n	Physical
Refractive index	μ	Physical
Sh.- & med.-range n.b. energy	E_{sm}	Physical
Solvent accessible reduct. ratio	R_a	Physical
Surrounding hydrophobicity	H_p	Physical
Thermodyn. transfer hydrophob.	H_t	Physical
Total n.b. energy	E_t	Physical
Turn tendencies	P	Physical
α−helical tendencies	P_α	Physical
β-structure tendencies	P_β	Physical

<u>Supplementary Table S4:</u> Paralytic and lethal effects of funnel-web spider venoms determined 1 h and 24 h after injection into adult sheep blowflies (*L. cuprina*). Table values are in µg/g.

**Species & (ID)**	**Gender**	**PD_{50}**		**LD_{50}**	
		**1h**	**24h**	**1h**	**24h**
H. cerberea **(sp.1)**	female	8.5	10.9	76.2	32.3
H. cerberea **(sp.3)**	female	7.5	11.4	173.6	52.2
H. infensa **(Orchid Beach, sp.5)**	female	10.2	5.9	61.0	17.8
H. infensa **(Mapleton Falls, sp.6)**	female	4.6	3.9	26.4	11.5
H. formidabilis **(Tamborine, sp.7)**	female	4.0	5.5	62.5	64.3
H. formidabilis **(Tamborine, sp.8)**	female	11.4	14.4	74.1	85.0
A. robustus **(P494)**	female	103.9	40.9	>500	>500
A. robustus **(P495)**	male	>500	57.5	>500	385.1
